# Sperm Migration and Hyaluronic Acid Binding: Implications for Male Fertility Evaluation

**DOI:** 10.3390/ijms25189995

**Published:** 2024-09-17

**Authors:** Katarzyna Marchlewska, Marta Erkiert-Kusiak, Renata Walczak-Jędrzejowska, Jolanta Słowikowska-Hilczer

**Affiliations:** 1 Division of Reproductive Endocrinology, Department of Andrology & Reproductive Endocrinology, Medical University of Lodz, Lodz 91-419, Poland; renata.walczak-jedrzejowska@umed.lodz.pl (R.W.-J.); jolanta.slowikowska-hilczer@umed.lodz.pl (J.S.-H.); 2 1st Department of Anesthesiology & Intensive Care, Medical University of Warsaw, Warsaw 02-005, Poland

**Keywords:** semen analysis, sperm motility, sperm function, HBA, sperm migration test, swim-up

## Abstract

Mature, vital, and motile spermatozoa are essential for reaching the oocyte and binding to hyaluronic acid (HA) in the cumulus oophorus matrix. This study aims to determine the relationship between sperm-migration ability and HA-binding potential, as well as the relationship between sperm concentration and motility. Semen samples were collected from 702 men aged 20–56 years (median 34.8). We evaluated the sperm concentration and motility from basic semen analysis, the swim-up test (expressed as millions per mL and the migration efficiency percentage), and the hyaluronan-binding assay (HBA). A moderate positive correlation was found between the migration test results and HBA (R = 0.48). The highest correlation was observed between the concentration of motile spermatozoa and the migration test results (R = 0.85) and HBA (R = 0.4). The sperm migration efficiency strongly correlated with progressive motility (R = 0.6). Although significantly higher sperm migration was observed in patients with normal HBA results, the results of the functional tests were found to differ in some cases. For infertility treatment, the current diagnostic algorithm should be enhanced with more comprehensive seminological methods that assess the sperm-migration ability and HA-binding potential. We also recommend incorporating the swim-up method into the diagnostic protocol before planning assisted reproductive technology (ART) treatment.

## 1. Introduction

Around 50% of couples experiencing conception difficulties are affected by male infertility [1]. Male fertility problems can be associated with impaired sperm migration in the female reproductive tract and/or impaired sperm–egg fusion. Spermatozoa, the final product of spermatogenesis, leave the testis as morphologically complete but functionally immature cells [2]. Their motility and final maturation are triggered by interactions with various factors present in the epididymis and further during their journey through the female reproductive tract [3]. Once in the cervical mucus, spermatozoa migrate to the uterine cavity and fallopian tubes, where capacitation is completed, making them competent to fertilize the oocyte [4].

Sperm recognize the location of the egg through a reaction with chemoattractants in a process called chemotaxis. The control of the sperm swimming path is regulated by a signaling pathway system in the flagellum. Although this process is well understood in animals that have external fertilization [5], it is difficult to study in mammals. However, numerous molecules have been proposed as sperm attractants, and it is likely that the sperm is directed by a combination of chemotaxis (chemical signaling) and thermotaxis (temperature gradients). Both the egg and its surrounding cumulus cells secrete sperm chemoattractants, which cause a transient rise in intracellular Ca^2+^ concentrations, leading to a sequence of membrane hyperpolarization and depolarization caused by the opposing actions of potassium channels and voltage-gated calcium channels. [6,7]. These sperm-specific calcium channels, called CatSper (cation channel of sperm), open during recovery from hyperpolarization under alkaline conditions [8]. The process of sperm chemotaxis is crucial for natural fertilization but is better established in marine invertebrates compared to humans. One candidate for a chemoattractant is progesterone [9,10], but a review of experimental studies shows a wide spectrum of substances being examined [11]. It has been shown that only a small portion of the sperm population (2–12%) is functionally mature and sensitive to chemoattraction by follicular factors [12]. Moreover, spermatozoa only acquire their chemotactic properties during capacitation [13]. It has been shown that analyzing selectively in vitro cultured cumulus cells as the chemoattractant source, instead of follicular fluid or progesterone-containing medium, attracts sperm [13].

Capacitation has been found to change motility patterns in sperm (hyperactivation) and prepare it for the acrosomal reaction, two events required for fertilization, both physiologically and in vitro. The process of capacitation is characterized by changes in the structure of the sperm membrane: its fluidity increases due to the action of albumin, which removes cholesterol, sterols, and non-covalently bound glycoproteins. This increase in membrane fluidity and permeability leads to an influx of bicarbonate and calcium ions into the sperm, triggering a cascade of signaling events that induce hyperactivation and prepare it for interaction with the egg [14,15,16,17]. The sperm plasma membrane contains chaperone molecules, also known as heat shock proteins (HSP), which play a role in protein folding and transport through membranes.

Particularly noteworthy is the HSPA2 protein, which affects sperm function. Together with two additional proteins, namely, sperm adhesion molecule 1 (SPAM1) and arylsulfatase A (ARSA), it forms a receptor complex that participates in the interaction between the sperm and the oocyte [18]. During migration through the fallopian tube, before capacitation, when the spermatozoa approach the oocyte, they encounter the hyaluronic acid (HA)-rich egg cumulus oophorus. The sperm membrane attaches to HA in the cumulus egg matrix via the SPAM1 protein expressed on its outer surface, causing it to disperse and allowing the sperm to penetrate the cumulus. Once this stage is completed, the sperm initiates capacitation and the HSPA2 protein undergoes a conformational change, resulting in the SPAM1 protein being transported to the inner surface of the membrane. This is followed by the expression of ARSA, the second protein of the receptor complex, which allows the sperm to adhere to the oocyte zona pellucida. The surface expression of these two proteins is regulated by the HSPA2 chaperone (Appendix A).

Although semen analysis is a crucial element in diagnosing male fertility, it does not differentiate between fertile and infertile males [19]. The male reproductive system produces a vast number of sperm daily, but only a small portion is functionally complete. It has been shown that only mature, vital, and motile spermatozoa can reach the oocyte and bind to hyaluronic acid (HA) in the cumulus oophorus matrix. The migration of spermatozoa can be assessed using a swim-up test; however, this test is usually reserved for isolating motile sperm, free of contaminants such as seminal plasma, cell debris, leukocytes, and bacteria, for various research or assisted reproductive technologies (ART). In such cases, different proportions of semen and medium are used to maximize the recovery of motile sperm [20,21,22]. The swim-up test also provides the possibility of predicting the number of spermatozoa that can migrate from the ejaculate. Under physiological conditions, these sperm are likely able to penetrate cervical mucus. This step of the fertilization process is no longer diagnosed, as human cervical mucus tests were eliminated from the new edition of the WHO manual and are no longer used in clinical practice [23]. These data are important when planning how to prepare sperm for ART procedures or to estimate the chances of success between intrauterine insemination (IUI) or in vitro fertilization (IVF). Male gametes may be separated from seminal plasma for different purposes and by various methods [23]. Clinically, the most important is sperm recovery for ART, indicating a strong need to supplement diagnostics with tests that assess semen quality in this context.

Additionally, the presence of only a few sperm capable of migration, especially when their total motility is normal, may suggest a distinct fertility problem. Immature sperm with impaired HA-binding ability and reduced HSPA2 expression are more likely to contain excessive cytoplasm and have a higher aneuploidy index [24,25,26].

The aim of this study was to determine the ability of sperm to migrate and bind to HA in men. It also examines the relationship between these two functions and their relationship to sperm concentration and motility. For the purpose of investigating the migration ability of sperm cells, we propose the swim-up test as a new diagnostic step.

## 2. Results

The baseline characteristics of the participants are summarized in Table 1. A normal spermatozoa concentration, according to WHO 2010, was observed in 77.7% of study participants, and sperm cells with normal progressive motility were observed in 64.1%. The median sperm concentration after the swim-up test (post-wash sperm count) was 4 × 10^6^/mL, and the median efficiency of the assay was 13.2%; in the native samples, 57% of sperm cells demonstrated total sperm motility, and 40% demonstrated progressive motility. The results of HBA ≥ 80% were recognized in 40.2% of the participants.

The correlation between the results of both functional assays and basic sperm parameters is shown in Table 2. The strongest relationship was found between TNP and TNM, as well as between the sperm concentration in the native sample and the results of the swim-up test, expressed as the sperm count (Table 2).

**Figure 1:**
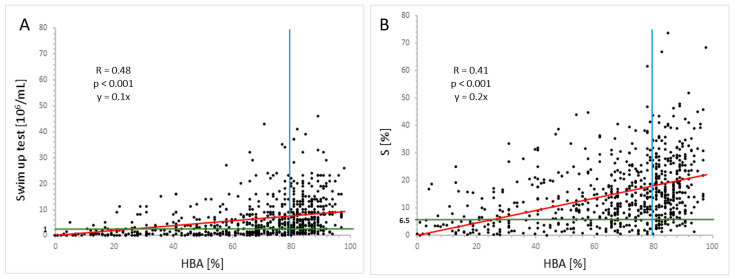
Correlation between the results of the HBA and (**A**) of the migration test, expressed as post-wash sperm count and (**B**) efficiency of sperm migration in the swim-up test (S). Blue line—reference value for HBA (Spearman’s R); green line—cut-off value for post-wash sperm count and S in the migration test, established specifically for this study; red line—trend estimation based on linear regression (trend line).

However, in the swim-up test, the efficiency of sperm migration correlated more closely with sperm motility than the sperm concentration. The ability of sperm to react with HA was found to be more closely associated with the migration test than with the analyzed basic semen parameters. The correlations with the results of the two assays are presented in Figure 1.

Although 40.2% (282 cases) of the study group had normal HBA results, 12% of these (34 cases) demonstrated migration efficiency <6.3% (the upper value of the lowest quartile). In those men with low migration efficiency, 3.6% (26 cases) exhibited a post-wash sperm count < 1 × 10^6^/mL (see the example in Appendix B). In contrast, 59.8% (420 participants) demonstrated HBA <80%; in this subgroup, around 33% demonstrated a post-wash sperm count (138 cases) or migration efficiency (139 cases) below the lower limit. Thus, more than 67% of participants with poor HBA results also demonstrated good spermatozoa migration (Table 3).

**Figure 2:**
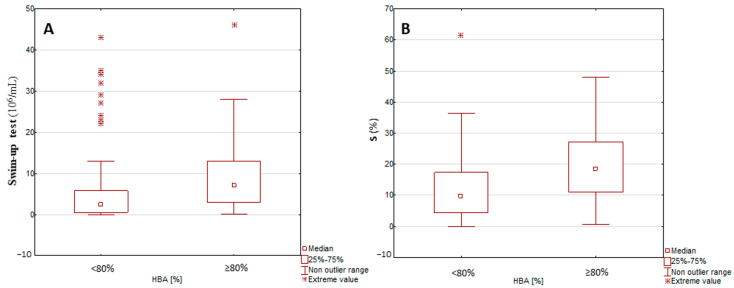
Results of (**A**) swim-up test (post-wash sperm count) and (**B**) efficiency of sperm migration in the swim-up test (S) in groups with normal and <80% HBA results. Mann–Whitney test (*p* < 0.0001).

Sperm migration was found to differ significantly between patients with normal and reduced HBA results (*p* < 0.0001) (Figure 2).

HBA results below reference values were associated with a significantly lower median post-wash sperm count and S-value in the swim-up test.

## 3. Discussion

The analysis of sperm migration in the female genital tract can provide insight into the factors determining sperm–egg fusion [16]. Standard sperm analysis can determine the percentage of sperm motility, but this does not accurately reflect the number of sperm that migrate during fertilization. While reports indicate that it is possible to diagnose normal or abnormal semen results by differentiating forms of male infertility based on WHO cut-off values [27], the authors emphasize that these values cannot be used to distinguish between fertile and subfertile men [19,23]. A better method, though still inadequate, is using a combination of basic sperm parameters such as the total motile sperm count [28,29,30]. Spermatozoa undergo various modifications during migration under physiological conditions; however, these cannot be accurately replicated in vitro [3,31]. For example, sperm analysis is performed before the cells leave the seminal fluid, which occurs during both natural and artificial fertilization and influences their function [4,32]; as such, it is difficult to predict which and how many sperm are capable of migration. Additionally, in vitro research results may depend on various factors, such as the composition of the media used in sperm-selection techniques. One component of the medium used in the migration test is albumin; contact with this medium allows spermatozoa to swim out of the seminal plasma, indicating the presence of positive chemotaxis. The chemotactic signaling pathway that causes chemoattractant-induced sperm migration in humans is still under review [33], but several attractants are used in experimental studies [34]. Our findings clearly show that not all motile spermatozoa are sensitive to chemotaxis. The difference is evident when comparing the median (range) result of TNM, which was 15.7 (0.4–163.3), and the result after swim-up, which was 4.0 (0.0–74), respectively (Table 1).

Our findings also indicate that sperm-migration ability is closely correlated with the concentration of motile cells. However, some patients demonstrate normal sperm motility but poor migration test results; these patients may have fewer sperm capable of reaching the oocyte in the oviduct, resulting in reduced natural fertility. This hypothesis requires further study in patients trying to conceive. Our results suggest that TNM and TMP have the strongest predictive value regarding sperm migration, showing the closest relationship with migration test results. In particular, the total motile sperm number is a widely analyzed parameter in native semen [28,29,30,35]. Our study shows that the efficiency of sperm migration (S) depends closely on progressive and total motility: no examples were observed where the number of sperm that migrated to the medium was equal to TNM. Some authors, however, believe that the number of sperm after preparation in the swim-up test has a higher predictive value [36,37,38].

The specific cut-off values for TNM and post-wash TNM are still the subject of discussion [36,39,40,41,42,43]. Although cut-off values were established in the swim-up test for this study, our findings are insufficient to determine reference values for the post-wash sperm count and the efficiency of sperm migration that correlate with fertility status; these should be considered in further research. Most studies on semen diagnostics try to establish threshold values for TNM or post-wash TNM, above which the fertilization rate increases. However, it seems impossible to identify an isolated factor that will have a decisive impact on such a complex system as fertilization. Instead, a better strategy would be to establish the values below which the fertilization process becomes unlikely or even impossible; this approach will better support decisions to undertake treatment and select an appropriate ART method. Therefore, it is worth performing a routine migration test before applying ART techniques such as intrauterine insemination (IUI). An interesting randomized, multicenter clinical trial showed that the chance of IUI resulting in a live birth was 5.5% with a post-wash TNM value ≤5 × 10^6^, while it rose to 14.8% for 15 to 20 × 10^6^ [44].

Even if the financial costs of IUI are not high, the procedure should still be sufficiently effective to merit consideration. If the expected fertilization rate is 10% or less [42,44,45], 90% of participating couples will still incur the cost and stress associated with this procedure without success.

One variable calculated specifically for this study was the efficiency of sperm migration in the swim-up test. This result indicates the percentage of sperm with the ability to migrate per 1 mL of a semen sample, which may be useful in predicting how many sperm are able to migrate from the entire ejaculate. The swim-up technique is only one of several sperm-processing methods. Meta-analyses have concluded that there is no significant difference in pregnancy rates following IUI with different semen-preparation techniques [46,47]. However, the migration test used in this study may be conducted in routine sperm analysis and provide clinical information on IUI effectiveness. It has been previously shown that the swim-up technique is very efficient in reducing the number of sperm with diminished maturity, as well as those with aneuploidies, diploidy, and DNA fragmentation [48,49,50,51]. It has been shown that the aneuploidy and diploidy rates and the frequency of chromosomally unbalanced spermatozoa were lower in the sperm bound to HA [49,52]. However, the S value is not strongly correlated with HA binding (R = 0.4), which may be explained by the fact that not all spermatozoa with the ability to migrate finally reach the oocyte. Of the total number of spermatozoa in ejaculate, only 10% will enter the cervix, 1% will enter the uterus, and 0.1% will enter the fallopian tube [53]. The efficiency of sperm migration may be important for predicting natural fertility potential, especially in patients with oligozoospermia, and it could be a useful parameter in diagnosing infertility causes. Such easy calculations provide additional information for planning an ART strategy. Additionally, information about the number of sperm that can be obtained during semen preparation is important when qualifying an infertile couple for IUI or IVF.

HBA is used to assess the ability of sperm to recognize the female gamete in vitro, more precisely HA, which is one of the components of the cumulus–oocyte complex. The interpretation of the test leads to a comparison with sperm chemotaxis assays based on increased sperm accumulation near the source of the chemoattractant [11]. The components of the cumulus have been used as chemoattractants in previous studies [13]. A lack of reaction between sperm and HA can indicate the absence of HSPA2, which can be connected with post-meiotic defects in spermatozoa maturation; low HSPA2 expression has been noted in immature spermatozoa with cytoplasmic retention, and high HSPA2 expression has been noted in mature spermatozoa without cytoplasmic retention [25]. Initially, HBA was invented to distinguish semen samples suitable for IVF or ICSI; however, it may also be used for selecting sperm without aneuploidy and DNA fragmentation for ICSI. However, the data regarding HA are inconsistent. Some authors report higher rates of fertilization and embryo quality [54,55], while others do not [56,57]. Another obstacle to interpreting the HBA-binding score is the cut-off value of the assay. The manufacturer established a limit value of 80%, indicating high sensitivity, but research data suggest much lower values like 65%, 60%, or even less [24,55,58], which may increase test specificity. It seems important to select spermatozoa from only male patients with truly abnormal HBA scores because the benefits of using PICSI randomly in infertile couples are limited [59]. In our data, almost 60% of participants presented results below the manufacturer’s cut-off limits; however, this percentage drops to 34% when the cut-off limit is set at 65%.

In recent years, the clinical value of the HBA in managing male infertility remains unresolved [60,61]. The process of receptor inhibition via endogenous and exogenous factors remains relatively unexplored, although a recent study found a potential contraceptive agent to induce HA receptor dysfunction [62]. It is possible that inter alia infections and iatrogenic factors with similar effects may be present in the male reproductive system, although this hypothesis needs further research.

## 4. Materials and Methods

### 4.1. Participants

A retrospective analysis was performed of semen samples obtained from 702 men, aged 20–56 years (median 34.8), who participated in two projects conducted in the Department of Andrology and Reproductive Endocrinology, Medical University of Lodz in the years 2012 to 2018. The participants recruited for the first study were men from the Outpatient’s Clinic of Andrology and Reproductive Endocrinology in Lodz. This group includes men whose fertility status has not been confirmed. The aim of this research project was to study the relationship between testicular germ cell neoplastic changes and sperm DNA damage together with disorders of spermatozoa functional maturation. The group did not include participants with testicular cancer or symptoms of infection.

Participants recruited for the second study included men from the general population with unknown fertility status. This study concerned the impact of lifestyle factors on sperm parameters. Patients with symptoms of infection were also excluded. Inclusion criteria were sperm concentrations >1 × 10^6^/mL and volume >1.3 mL. All participants received detailed information about the study and provided their written consent to take part.

The study was approved by the Bioethical Committee of the Medical University in Lodz, Poland (No. RNN/347/15/KE and RNN/125/12/KE).

### 4.2. Basic Semen Analysis

The basic semen analysis was performed according to WHO [27] recommendations from 2010 by a single technician who regularly (twice a year) participated in external quality control (QuaDeGa GmbH, quality control scheme of the German Society of Andrology; https://www.quadega.de/ accessed on 1 July 2024). Semen samples were collected via masturbation after two to seven days of sexual abstinence in pre-weighed, disposable containers. Microscopic evaluation was performed with a phase-contrast microscope (Nikon Eclipse E600, Tokyo, Japan) at a magnification of 400×. Sperm motility was assessed just after liquefaction (no longer than 60 min after ejaculation). The spermatozoa with progressive or non-progressive motility were distinguished from immotile cells. Sperm concentration was evaluated using a Neubauer hemocytometer. Values of progressive motility ≥32%, total motility ≥40%, and sperm concentration ≥15 × 10^6^/mL were considered normal.

Two additional parameters were calculated for the native semen: total number of sperm with progressive motility (TNP)/1 mL and total number of motile sperm (TNM)/1 mL.

### 4.3. Assessment of Spermatozoa Migration with Swim-Up Test

Spermatozoa are separated from seminal plasma for a variety of purposes, such as tests for functional competency, evaluating the effects of media composition, and sperm recovery in ART [63,64,65]. In the swim-up technique, gametes are selected by their ability to swim out of seminal plasma and into culture medium. However, although the results obtained after preparation support the interpretation of further clinical procedures, no diagnostic test has yet been developed.

In the swim-up test, spermatozoa migrate to QUINN’S Sperm Washing Medium, (SAGE Media, Ballerup, Denmark). A pH (7.2–7.4), ionic, and protein composition similar to human tubal fluid is obtained using a buffering system: 21 mM of HEPES (N-2-Hydroxyethylpiperazine-N1-2-ethanesulfonic acid) and 4 mM of sodium bicarbonate and human albumin at a concentration of 5 mg/mL.

Exactly 1 mL of a washing solution warmed to 37 °C was pipetted into a Falcon tube, and 1 mL of well-mixed semen sample was carefully added to the medium. As the specific density of the semen is greater than that of the washing solution, two non-miscible phases were obtained: the lower, containing semen, and the upper, containing medium. The semen sample was incubated for 60 min in an incubator (Melag, Incubat, Berlin Germany) at 37 °C at an angle of 45°. After 60 min, the supernatant with motile spermatozoa was aspirated into Eppendorf tubes using a pipette (centrifugation step was skipped); after mixing, the sperm concentration was assessed, which was considered as the test result. Additionally efficiency of sperm migration in the swim-up test (S) was calculated using the following formula:

$$S=\frac{sperm\;concentration\;after\;swim-up\times 100\%}{sperm\;concentration\;in\;native\;semen}$$

The swim-up test is not a typical diagnostic assay; as such, there is no established lower limit as a reference value. In this study, the lower quartile of obtained results was used as a cut-off value; thus, the lower quartile limit was 1.0 × 10^6^/mL for the post-wash sperm count and 6.3% for S in the swim-up test.

### 4.4. Sperm Binding to Hyaluronic Acid

The Hyaluronan Binding Assay (HBA) (Biocoat Inc., Horsham, PA, USA) was used to determine the proportion of sperm with the ability to bind with HA. The assay was carried out at room temperature. Each semen sample was mixed thoroughly using plastic disposable pipettes, and 10 µL was pipetted on the center of a special chamber coated with solid-state hyaluronan (supplied with the kit). A CELL-VU-gridded cover slip was placed over the chamber, avoiding air bubble formation. The chamber was incubated at room temperature for 10–20 min.

The samples were analyzed with a phase-contrast microscope (Nikon Eclipse E600, Tokyo, Japan) at a magnification of 400×. The numbers of bound motile spermatozoa and total motile spermatozoa were scored. The immotile spermatozoa were not taken into consideration. The ratio of HA-bound motile spermatozoa to all motile spermatozoa was calculated and shown as a percentage. The percentage of HA-bound sperm ≥80% was considered as normal, as recommended by the manufacturer of the assay.

All analyses were performed in two replicates. Approximately 200 spermatozoa per replicate were assessed.

### 4.5. Statistics

All analyses were performed using Statistica 13.1 for Windows (StatSoft Inc., Tulsa, OK, USA). The distribution of the data was analyzed using the Shapiro–Wilk test. The data were distributed in a nonparametric manner; therefore, the data were presented as median and interquartile range, and the groups were compared using the Mann–Whitney test. The sperm parameters were also compared using Spearman’s rank correlation test. Reliability value is interpreted based on Guilford’s Reliability Coefficient Classification. R < 0.2 indicates a lack of linear dependence, R in the range of >0.2–0.4 indicates weak dependence, R in the range of >0.4–0.7 indicates moderate dependence, R in the range of >0.7–0.9 indicates strong dependence, and R > 0.9 indicates very strong dependence. Differences were considered significant at *p* < 0.05.

## 5. Conclusions

Our research highlights the importance of incorporating tests that assess sperm migration and HA-binding capacity into seminological diagnostics when selecting a treatment strategy for infertile couples. While these tests serve as pharmacokinetic biomarkers and are not suitable for evaluating the effectiveness of assisted reproductive technologies (ART), they may play a crucial role in guiding the choice of appropriate therapeutic procedure.

## Figures and Tables

**Table 1:** Baseline characteristics of the study group (n = 702).

Parameter	Mean (±SD)	Median	Range
Age (years)	34.8 (±5.3)	34.0	20–56
Semen volume (mL)	3.9 (±1.7)	3.6	1.3–9.3
Sperm concentration (10^6^/mL)	37.6 (±33.0)	27.0	1–280
Progressive motility (%)	39.0 (±14.2)	40.0	1.0–81.0
Total motility (%)	55.4 (±13.5)	57.0	5.0–88.0
TNP (10^6^/mL)	15.6 (±15.4)	10.5	0.1–119.6
TNM (10^6^/mL)	21.3 (±19.7)	15.7	0.4–163.3
Swim-up (10^6^/mL)	6.4 (±1.3)	4.0	0.0–74.0
S (%)	15.3 (±1.9)	13.2	0–73.5
HBA (%)	67.1 (±22.9)	74.0	0.0–98.0

Abbreviations: TNM—total number of motile sperm in 1 mL of native semen; TNP—total number of sperm with progressive motility in 1 mL of native semen; S—efficiency of sperm migration in the swim-up test; SD—standard deviation.

**Table 2:** Correlations between the results of the swim-up test, efficiency of sperm migration in the swim-up test (**S**), and HBA (% sperm bound with hyaluronic acid) with sperm concentration and motility (Spearman’s R). All presented coefficients are statistically significant (*p* < 0.05).

	Swim-Up Test (10^6^/mL)	S (%)	HBA (%)
Sperm concentration (10^6^/mL)	0.78	0.29	0.36
Progressive motility (%)	0.55	0.61	0.30
Total motility (%)	0.45	0.58	0.23
TNP (10^6^/mL)	0.87	0.50	0.41
TNM (10^6^/mL)	0.86	0.44	0.40

Abbreviations: TNM—total number of motile sperm in 1 mL of native semen; TNP—total number of sperm with progressive motility in 1 mL of native semen.

**Table 3:** The results of the swim-up test, efficiency of sperm migration in the swim-up test (**S**), and HBA (% sperm bound with hyaluronic acid) according to cut-off limits.

	Number of Cases (n = 702)	%
HBA ≥ 80% and Swim-up test ≥ 1 × 10^6^/mL	261	37
HBA ≥ 80% and Swim-up test < 1 × 10^6^/mL	21	3
HBA < 80% and Swim-up test ≥ 1 × 10^6^/mL	277	40
HBA < 80% and Swim-up test < 1 × 10^6^/mL	143	20
HBA ≥ 80% and S ≥ 6.3%	248	35
HBA ≥ 80% and S < 6.3%	34	5
HBA < 80% and S ≥ 6.3%	279	40
HBA < 80% and S < 6.3%	141	20

**Figure A1:**
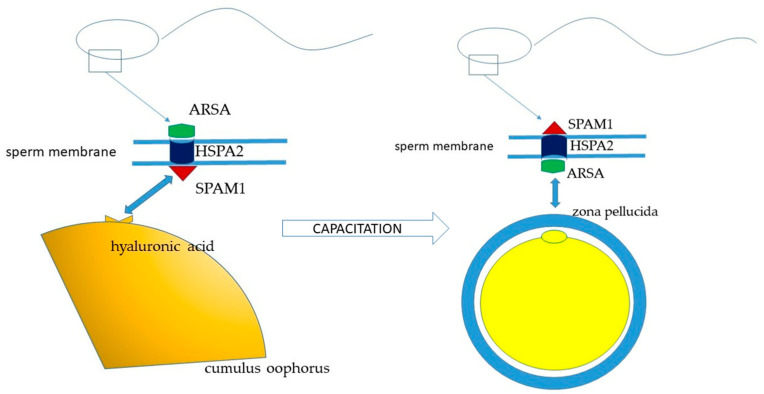
The changes occurring in the hyaluronic acid-binding receptor during the capacitation process.

## Data Availability

The data presented in this study are available upon reasonable request from the corresponding author.

## Appendix B

**Table A1:** The example results of two patients whose sperm showed a relatively high percent of sperm binding to HA and normal sperm concentration, but the ability to migrate was very low.

Initials (age years)	Parameter	Value	Unit of Measure
MP (29)	Sperm concentration	81	10^6^/mL
	Progressive motility	22	%
	Total motility	47	%
	TNP	17.8	10^6^/mL
	TNM	38.1	10^6^/mL
	HBA	83	%
	Swim-up test	0.4	10^6^/mL
	S	0.5	%
TK (36)	Sperm concentration	21	10^6^/mL
	Progressive motility	39	%
	Total motility	56	%
	TNP	8.2	10^6^/mL
	TNM	11.8	10^6^/mL
	HBA	78	%
	Swim-up test	0.1 *	10^6^/mL
	S	0.5	%

Abbreviations: TNM—total number of motile sperm in 1 mL of native semen; TNP—total number of sperm with progressive motility in 1 mL of native semen. *—(<0.1) approximate value below the sensitivity of the counting chamber.

## References

[1] Agarwal A., Baskaran S., Parekh N., Cho C.L., Henkel R., Vij S., Arafa M., Panner Selvam M.K., Shah R. (2021). Male infertility. Lancet.

[2] Baldi E., Luconi M., Bonaccorsi L., Forti G. (2002). Signal transduction pathways in human spermatozoa. J. Reprod. Immunol..

[3] Freitas M.J., Vijayaraghavan S., Fardilha M. (2017). Signaling mechanisms in mammalian sperm motility. Biol. Reprod..

[4] Suarez S.S., Pacey A.A. (2006). Sperm transport in the female reproductive tract. Hum. Reprod. Update.

[5] Friedrich B.M., Julicher F. (2007). Chemotaxis of sperm cells. Proc. Natl. Acad. Sci. USA.

[6] Bohmer M., Van Q., Weyand I., Hagen V., Beyermann M., Matsumoto M., Hoshi M., Hildebrand E., Kaupp U.B. (2005). Ca2+ spikes in the flagellum control chemotactic behavior of sperm. EMBO J..

[7] Strunker T., Weyand I., Bonigk W., Van Q., Loogen A., Brown J.E., Kashikar N., Hagen V., Krause E., Kaupp U.B. (2006). A K+-selective cGMP-gated ion channel controls chemosensation of sperm. Nat. Cell Biol..

[8] Seifert R., Flick M., Bonigk W., Alvarez L., Trotschel C., Poetsch A., Muller A., Goodwin N., Pelzer P., Kashikar N.D. (2015). The CatSper channel controls chemosensation in sea urchin sperm. EMBO J..

[9] Teves M.E., Guidobaldi H.A., Unates D.R., Sanchez R., Miska W., Publicover S.J., Morales Garcia A.A., Giojalas L.C. (2009). Molecular mechanism for human sperm chemotaxis mediated by progesterone. PLoS ONE.

[10] Jaiswal B.S., Tur-Kaspa I., Dor J., Mashiach S., Eisenbach M. (1999). Human sperm chemotaxis: Is progesterone a chemoattractant?. Biol. Reprod..

[11] Burnett L.A., Washburn C.A., Sugiyama H., Xiang X.Y., Olson J.H., Al-Anzi B., Bieber A.L., Chandler D.E. (2012). Allurin, an Amphibian Sperm Chemoattractant Having Implications for Mammalian Sperm Physiology. Int. Rev. Cel. Mol. Bio..

[12] CohenDayag A., TurKaspa I., Dor J., Mashiach S., Eisenbach M. (1995). Sperm capacitation in human is transient and correlates with chemotactic responsiveness. Colloq. Inse..

[13] Xie L., Ma R., Han C., Su K., Zhang Q.F., Qiu T.A., Wang L., Huang G.L., Qiao J., Wang J.D. (2010). Integration of Sperm Motility and Chemotaxis Screening with a Microchannel-Based Device. Clinical. Chem..

[14] Reid A.T., Redgrove K., Aitken R.J., Nixon B. (2011). Cellular mechanisms regulating sperm-zona pellucida interaction. Asian J. Androl..

[15] Breitbart H., Etkovitz N. (2011). Role and regulation of EGFR in actin remodeling in sperm capacitation and the acrosome reaction. Asian J. Androl..

[16] Ickowicz D., Finkelstein M., Breitbart H. (2012). Mechanism of sperm capacitation and the acrosome reaction: Role of protein kinases. Asian J. Androl..

[17] Cohen R., Mukai C., Travis A.J. (2016). Lipid Regulation of Acrosome Exocytosis. Adv. Anat. Embryol. Cell Biol..

[18] Redgrove K.A., Nixon B., Baker M.A., Hetherington L., Baker G., Liu D.Y., Aitken R.J. (2012). The molecular chaperone HSPA2 plays a key role in regulating the expression of sperm surface receptors that mediate sperm-egg recognition. PLoS ONE.

[19] Bjorndahl L. (2011). What is normal semen quality? On the use and abuse of reference limits for the interpretation of semen analysis results. Hum. Fertil..

[20] Oguz Y., Guler I., Erdem A., Mutlu M.F., Gumuslu S., Oktem M., Bozkurt N., Erdem M. (2018). The effect of swim-up and gradient sperm preparation techniques on deoxyribonucleic acid (DNA) fragmentation in subfertile patients. J. Assist. Reprod. Genet..

[21] Muratori M., Tarozzi N., Carpentiero F., Danti S., Perrone F.M., Cambi M., Casini A., Azzari C., Boni L., Maggi M. (2019). Sperm selection with density gradient centrifugation and swim up: Effect on DNA fragmentation in viable spermatozoa. Sci. Rep..

[22] Holt W.V., Hernandez M., Warrell L., Satake N. (2010). The long and the short of sperm selection in vitro and in vivo: Swim-up techniques select for the longer and faster swimming mammalian sperm. J. Evol. Biol..

[23] WHO (2021). WHO Laboratory Manual for the Examination and Processing of Human Semen.

[24] Huszar G., Ozenci C.C., Cayli S., Zavaczki Z., Hansch E., Vigue L. (2003). Hyaluronic acid binding by human sperm indicates cellular maturity, viability, and unreacted acrosomal status. Fertil. Steril..

[25] Cayli S., Jakab A., Ovari L., Delpiano E., Celik-Ozenci C., Sakkas D., Ward D., Huszar G. (2003). Biochemical markers of sperm function: Male fertility and sperm selection for ICSI. Reprod. Biomed. Online.

[26] Huszar G., Jakab A., Sakkas D., Ozenci C.C., Cayli S., Delpiano E., Ozkavukcu S. (2007). Fertility testing and ICSI sperm selection by hyaluronic acid binding: Clinical and genetic aspects. Reprod. Biomed. Online.

[27] WHO (2010). WHO Laboratory Manual for the Examination and Processing of Human Semen.

[28] Borges E., Setti A.S., Braga D.P., Figueira R.C., Iaconelli A. (2016). Total motile sperm count has a superior predictive value over the WHO 2010 cut-off values for the outcomes of intracytoplasmic sperm injection cycles. Andrology.

[29] Hamilton J.A., Cissen M., Brandes M., Smeenk J.M., de Bruin J.P., Kremer J.A., Nelen W.L., Hamilton C.J. (2015). Total motile sperm count: A better indicator for the severity of male factor infertility than the WHO sperm classification system. Hum. Reprod..

[30] Tiegs A.W., Landis J., Garrido N., Scott R.T., Hotaling J.M. (2019). Total Motile Sperm Count Trend Over Time: Evaluation of Semen Analyses From 119,972 Men From Subfertile Couples. Urology.

[31] De Jonge C. (2017). Biological basis for human capacitation-revisited. Hum. Reprod. Update.

[32] Suarez S.S. (2016). Mammalian sperm interactions with the female reproductive tract. Cell Tissue Res..

[33] Hildebrand E., Kaupp U.B. (2005). Sperm chemotaxis: A primer. Ann. N. Y. Acad. Sci..

[34] Kaupp U.B., Kashikar N.D., Weyand I. (2008). Mechanisms of sperm chemotaxis. Annu. Rev. Physiol..

[35] Ombelet W., Dhont N., Thijssen A., Bosmans E., Kruger T. (2014). Semen quality and prediction of IUI success in male subfertility: A systematic review. Reprod. Biomed. Online.

[36] Dinelli L., Courbiere B., Achard V., Jouve E., Deveze C., Gnisci A., Grillo J.M., Paulmyer-Lacroix O. (2014). Prognosis factors of pregnancy after intrauterine insemination with the husband’s sperm: Conclusions of an analysis of 2,019 cycles. Fertil. Steril..

[37] Merviel P., Heraud M.H., Grenier N., Lourdel E., Sanguinet P., Copin H. (2010). Predictive factors for pregnancy after intrauterine insemination (IUI): An analysis of 1038 cycles and a review of the literature. Fertil. Steril..

[38] Berg U., Brucker C., Berg F.D. (1997). Effect of motile sperm count after swim-up on outcome of intrauterine insemination. Fertil. Steril..

[39] Van Voorhis B.J., Barnett M., Sparks A.E., Syrop C.H., Rosenthal G., Dawson J. (2001). Effect of the total motile sperm count on the efficacy and cost-effectiveness of intrauterine insemination and in vitro fertilization. Fertil. Steril..

[40] Berker B., Sukur Y.E., Kahraman K., Atabekoglu C.S., Sonmezer M., Ozmen B., Ates C. (2012). Absence of rapid and linear progressive motile spermatozoa "grade A" in semen specimens: Does it change intrauterine insemination outcomes?. Urology.

[41] Dorjpurev U., Kuwahara A., Yano Y., Taniguchi T., Yamamoto Y., Suto A., Tanaka Y., Matsuzaki T., Yasui T., Irahara M. (2011). Effect of semen characteristics on pregnancy rate following intrauterine insemination. J. Med. Invest..

[42] Nikbakht R., Saharkhiz N. (2011). The influence of sperm morphology, total motile sperm count of semen and the number of motile sperm inseminated in sperm samples on the success of intrauterine insemination. Int. J. Fertil. Steril..

[43] Zadehmodarres S., Oladi B., Saeedi S., Jahed F., Ashraf H. (2009). Intrauterine insemination with husband semen: An evaluation of pregnancy rate and factors affecting outcome. J. Assist. Reprod. Genet..

[44] Hansen K.R., Peck J.D., Coward R.M., Wild R.A., Trussell J.C., Krawetz S.A., Diamond M.P., Legro R.S., Coutifaris C., Alvero R. (2020). Intrauterine insemination performance characteristics and post-processing total motile sperm count in relation to live birth for couples with unexplained infertility in a randomised, multicentre clinical trial. Hum. Reprod..

[45] Muthigi A., Jahandideh S., Bishop L.A., Naeemi F.K., Shipley S.K., O’Brien J.E., Shin P.R., Devine K., Tanrikut C. (2021). Clarifying the relationship between total motile sperm counts and intrauterine insemination pregnancy rates. Fertil. Steril..

[46] Cil N., Kabukcu C., Cabus U., Turan T., Mete C.A. (2022). Retrospective comparison of the semen preparation techniques for intrauterine insemination: Swim-up versus density gradient method. J. Gynecol. Obstet. Hum..

[47] Boomsma C.M., Cohlen B.J., Farquhar C. (2019). Semen preparation techniques for intrauterine insemination. Cochrane Db Syst. Rev..

[48] Jakab A., Kovacs T., Zavaczki Z., Borsos A., Bray-Ward P., Ward D., Huszar G. (2003). Efficacy of the swim-up method in eliminating sperm with diminished maturity and aneuploidy. Hum. Reprod..

[49] Vozdova M., Kasikova K., Oracova E., Prinosilova P., Rybar R., Horinova V., Gaillyova R., Rubes J. (2012). The effect of the swim-up and hyaluronan-binding methods on the frequency of abnormal spermatozoa detected by FISH and SCSA in carriers of balanced chromosomal translocations. Hum. Reprod..

[50] Kim S.W., Jee B.C., Kim S.K., Kim S.H. (2017). Sperm DNA fragmentation and sex chromosome aneuploidy after swim-up versus density gradient centrifugation. Clin. Exp. Reprod. Med..

[51] Le M.T., Dang H.N.T., Nguyen T.V., Nguyen T.T.T., Nguyen Q.H.V., Cao N.T. (2022). Effects of sperm preparation techniques on sperm survivability and DNA fragmentation. J. Int. Med. Res..

[52] Jakab A., Sakkas D., Delpiano E., Cayli S., Kovanci E., Ward D., Ravelli A., Huszar G. (2005). Intracytoplasmic sperm injection: A novel selection method for sperm with normal frequency of chromosomal aneuploidies. Fertility and Sterility.

[53] Williams M., Hill C.J., Scudamore I., Dunphy B., Cooke I.D., Barratt C.L. (1993). Sperm numbers and distribution within the human fallopian tube around ovulation. Hum. Reprod..

[54] Parmegiani L., Cognigni G.E., Bernardi S., Troilo E., Ciampaglia W., Filicori M. (2010). "Physiologic ICSI": Hyaluronic acid (HA) favors selection of spermatozoa without DNA fragmentation and with normal nucleus, resulting in improvement of embryo quality. Fertil. Steril..

[55] Mokanszki A., Tothne E.V., Bodnar B., Tandor Z., Molnar Z., Jakab A., Ujfalusi A., Olah E. (2014). Is sperm hyaluronic acid binding ability predictive for clinical success of intracytoplasmic sperm injection: PICSI vs. ICSI?. Syst. Biol. Reprod. Med..

[56] Van Den Bergh M.J., Fahy-Deshe M., Hohl M.K. (2009). Pronuclear zygote score following intracytoplasmic injection of hyaluronan-bound spermatozoa: A prospective randomized study. Reprod. Biomed. Online.

[57] Kovacs P., Kovats T., Sajgo A., Szollosi J., Matyas S., Kaali S.G. (2011). The role of hyaluronic acid binding assay in choosing the fertilization method for patients undergoing IVF for unexplained infertility. J. Assist. Reprod. Genet..

[58] Worrilow K.C., Eid S., Woodhouse D., Perloe M., Smith S., Witmyer J., Ivani K., Khoury C., Ball G.D., Elliot T. (2013). Use of hyaluronan in the selection of sperm for intracytoplasmic sperm injection (ICSI): Significant improvement in clinical outcomes--multicenter, double-blinded and randomized controlled trial. Hum. Reprod..

[59] Miller D., Pavitt S., Sharma V., Forbes G., Hooper R., Bhattacharya S., Kirkman-Brown J., Coomarasamy A., Lewis S., Cutting R. (2019). Physiological, hyaluronan-selected intracytoplasmic sperm injection for infertility treatment (HABSelect): A parallel, two-group, randomised trial. Lancet.

[60] Tarozzi N., Nadalini M., Bizzaro D., Serrao L., Fava L., Scaravelli G., Borini A. (2009). Sperm-hyaluronan-binding assay: Clinical value in conventional IVF under Italian law. Reprod. Biomed. Online.

[61] West R., Coomarasamy A., Frew L., Hutton R., Kirkman-Brown J., Lawlor M., Lewis S., Partanen R., Payne-Dwyer A., Roman-Montanana C. (2022). Sperm selection with hyaluronic acid improved live birth outcomes among older couples and was connected to sperm DNA quality, potentially affecting all treatment outcomes. Hum. Reprod..

[62] North B.B., Weitzel M.B., Waller D.P., Birch W.X., Feathergill K.A., Birch L.A., De Jonge C.J., Prins G.S. (2022). Evaluation of the novel vaginal contraceptive agent PPCM in preclinical studies using sperm hyaluronan binding and acrosome status assays. Andrology.

[63] Henkel R.R., Schill W.B. (2003). Sperm preparation for ART. Reprod. Biol. Endocrinol..

[64] Henkel R. (2012). Sperm preparation: State-of-the-art--physiological aspects and application of advanced sperm preparation methods. Asian J. Androl..

[65] Mehta A., Sigman M. (2014). Identification and preparation of sperm for ART. Urol. Clin. North. Am..

